# Larch Wood Residues Valorization through Extraction and Utilization of High Value-Added Products

**DOI:** 10.3390/polym12020359

**Published:** 2020-02-06

**Authors:** Kerstin Wagner, Maurizio Musso, Stefan Kain, Stefan Willför, Alexander Petutschnigg, Thomas Schnabel

**Affiliations:** 1 Department of Forest Products Technology & Timber Constructions, Salzburg University of Applied Sciences, Marktstraße 136a, 5431 Kuchl, Austria; kerstin.wagner@stud.sgb.ac.at (K.W.); stefan.kain@fh-salzburg.ac.at (S.K.); alexander.petutschnigg@fh-salzburg.ac.at (A.P.); 2 Salzburg Center for Smart Materials, c/o Department of Chemistry and Physics of Materials, Paris Lodron University of Salzburg, Jakob-Haringer-Strasse 2A, 5020 Salzburg, Austria; 3 Department of Chemistry and Physics of Materials, Paris Lodron University of Salzburg, Jakob-Haringer-Strasse 2A, 5020 Salzburg, Austria; maurizio.musso@sbg.ac.at; 4 Laboratory of Natural Materials Technology, Åbo Akademi University, Porthasgatan 3, 20500 Turku/Åbo, Finland; stefan.willfor@abo.fi; 5 Department of Material Sciences and Process Engineering, BOKU University of Natural Resources and Life Sciences, Konrad Lorenz-Straße 24, 3340 Tulln, Austria

**Keywords:** GC-MS, kaempferol, knotwood, larixol, taxifolin, vibrational spectroscopy

## Abstract

Many of current bio-based materials are not fully or partly used for material utilization, as the composition of their raw materials and/or possible applications are unknown. This study deals with the analysis of the wood extractives from three different tissue of larch wood: Sapwood mainly from outer part of the log, and sound knotwood as well as dead knotwood. The extractions were performed with an accelerated solvent extractor (ASE) using hexane and acetone/water. The obtained extracts were analyzed by gas chromatography coupled to mass spectrometry (GC-MS). Three various vibrational spectroscopy (FT-RAMAN, FT-IR and FT-NIR) methods reflect the information from the extracts to the chemical composition of the types of wood before the extraction processes. Multivariate data analysis of the spectra was used to obtain a better insight into possible classification methods. Taxifolin and kaempferol were found in larger amount in sound knotwood samples compared to larch wood with high percentage of sapwood and dead knotwood samples. While the extractions of dead knotwood samples yielded more larixol and resin acids than the other larch wood samples used. Based on the chemical composition, three lead compounds were defined for the classification of the different wood raw materials. The vibrational spectroscopy methods were applied to show their potential for a possible distinction of the three types of larch wood tissue. This new insight into the different larch wood extracts will help in the current efforts to use more environmentally friendly raw materials for innovative applications. The connection between the raw materials and extraction yields of the target values is important to transform the results from the laboratory to industry and consumer applications.

## 1. Introduction

Regarding the bio-economy and bio-refinery approach, not all substances in the wood are currently used. New thinking and techniques are helpful to exploit the huge potential of bio-based products for innovative applications from the laboratory to industry. Different gaps exist and bridges are necessary to use the by-products (e.g., bark) for new applications (e.g., bio-based polymers) and not only for energy generation [1,2,3,4]. This study deals with the analysis of wood chips from an industrial Austrian sawmill for the potential uses of wood extractives.

The European larch (*Larix decidua* Mill.) is a commercially important species in the European Alpine areas and larch wood chips are normally produced as one of the by-products during the sawing process of wood. The outer part (mostly of it is sapwood) of the timber round wood cannot be used for the sawn wood and it is chopped to wood chips and used as thermal energy resources in larger part. As larch wood is not a preferred pulpwood species [5], since the amounts of resin and other extractives are large [6,7]. Furthermore, there is a non-negligible amount of knots in the fraction of these wood chips, which are sorted out during the pulp and paper process. However, knots contain exceptionally large amounts of secondary plant metabolites (e.g., phenolic compounds). The amount of knot wood extracts is often higher than in the stemwood for many tree species [8,9,10], and the hydrophilic compounds exhibit strong bioactivity against oxidation, bacteria and fungi exploitable for various new applications (e.g., dietary supplements and cosmetic products) [8,11,12,13].

**Figure 1:**
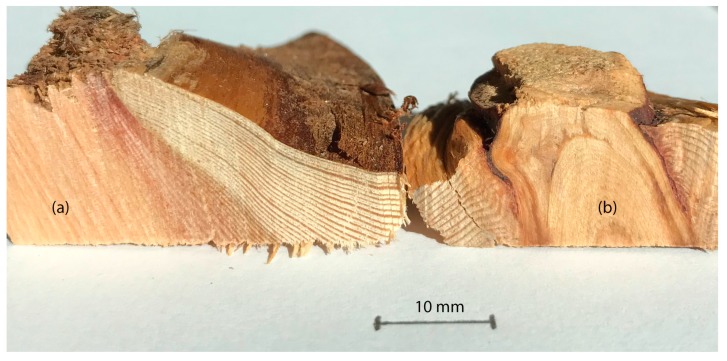
(**a**) One example of a larch wood chip with a sound knot and the intact connection between knot and stem wood; (**b**) a larch wood chip with a dead knot and the inclusion of bark as well as oxidized resin.

A knot or knotwood is indicated as the part of the branch which starts to grow from the pith and is hidden inside the stem. Two different types of knots can be distinguished, based on their function (Figure 1). Sound knots are connected with the stem wood and intact branches of the tree. If the lifetime of the branch is over then the tree loses the branches; however, the knot will be protected against bacteria and fungi by the wood extractives [14]. This knot is called a dead knot. As the stem grows further, it will gradually grow over the stump of lost branches, and finally the knot will be completely embedded in the stem wood (cf. Figure 1b).

The chemical composition of sound and dead knots of different larch wood species has been analyzed by Nisula [15]. As polyphenols are responsible for different functions of wood extracts (e.g., antimicrobial activities) [8,12,13], the results of chemical analysis of different parts of the trees are interesting for discovering wood tissues with a large extraction yield. The difference between both knot types was not focused upon in various studies of extractives and material properties [8,9,10,13]. GC-MS analyses are often applied to determine the chemical composition of wood extractives [6,8,9,10,12,13]. This method gives reliable results and is used for the detection of single components of natural extracts. However, in some processes less time-consuming analytical methods are important for the identification of different materials. Vibrational spectroscopy methods have much potentials for the sensitive and selective determination of organic compounds in wood and wood extractives [16,17,18]. These methods are also relatively inexpensive to implement and easy to apply; furthermore, they are non-destructive techniques [19].

In this study, the qualitative and quantitative chemical differences of sound and dead knots extracts were analyzed using a GC-MS method. New insight into the different extracts contents of bio-based polymers in natural resources will help in the current efforts to use more environmentally friendly raw materials for innovative applications. Based on the chemical composition, three lead compounds were defined for the classification of the different wood raw materials. Furthermore, FT-RAMAN, FT-IR and FT-NIR vibrational spectroscopy methods were applied for development of classification between sound and dead knots, which is also needed for the transformation of the results from the laboratory to the industry. The connection between raw materials and extraction yields of the target values is important for the utilization of different current unused wood tissues.

## 2. Materials and Methods

### 2.1. Wood Material

The wood chips of heart and sapwood from the outer part of the timber round wood as well as sound and dead knots from European larch (*Larix decidua* Mill.) trees were collected from an Austrian larch sawmill and ground with a cut mill (Retsch) using solid carbon dioxide to pass a mesh of 500 µm. The wood powder was then freeze-dried.

### 2.2. Chemicals

Taxifolin and kaempferol were produced by Alfa Aesar and Cayman, respectively, were used as reference.

### 2.3. Solid-Liquide Extraction

The extraction was done with an ASE (Accelerated Solvent Extractor) according to Willför [10]. The larch was first extracted with hexane (solvent temperature 90 °C, pressure 13.8 MPa, 2 × 5 min static cycles) and then with acetone (VWR) and water in a ratio 95/5 mixture (100 °C, 13.8 MPa, 2 × 5 min).

### 2.4. GC-MS Characterization

Before GC-MS analysis, the different extractives were evaporated using nitrogen gas and silylated to enhance volatility. For silylation, the evaporated extractives were first dried in a vacuum oven at 40 °C (Binder, Herbertshausen, Germany) and then silylation solvents (80 µL bis-(trimethylsilyl)-trifluoroacetamide, 20 µL pyridine and 20 µL trimethylsilyl-chloride) were added. Finally, the samples were incubated at 70 °C for 45 min. Measurements were performed using a Perkin Elmer Auto-System XL gas chromatograph (GC; PerkinElmer Inc., Waltham, MA, USA) and a GC-MS (HP 6890-5973 from Agilent Technologies Inc., Santa Clara, CA, USA). The GC was equipped with a HP-5 column (length: 25 m; ID: 0.20 mm; film thickness 0.11 µm) and a flame ionization detector (FID). The carrying gas was nitrogen at a flow rate of 0.8 mL/min. Furthermore, other conditions were: internal oven 120 °C with an increasing rate at 6 °C/min to 320 °C (15 min hold); a split injection with a ratio of 25:1 and a temperature of 250 °C; the detector temperature 310 °C and injection volume of 1 µL. The data were analysed based on the mass spectra library created at the Laboratory of Natural Materials Tehcnology at Åbo Akademi University.

### 2.5. Fourier Transform-RAMAN Spectroscopy

The wood powders (cf. Section 2.1) of different materials were examined in the NIR spectral region by FT-RAMAN using an IFS 66 FT-IR spectrometer (Bruker, Ettlingen, Germany) equipped with a Raman module FRA 106 and having a laser source at 1064 nm and a maximum power of 100 mW. The spectra were taken in the wavenumber range of 3500–100 cm^−1^ with a FWHM-resolution of 4 cm^−1^ and 400 cumulated scans.

### 2.6. Fourier Transform Infrared Spectroscopy

Small thin sliced samples of the various parts of the wood were produced and measured. The FT-IR spectra of different solid wood parts were recorded between 4000 and 600 cm^−1^ with 32 scans at a resolution of 4 cm^−1^ using a Frontier FT-IR spectrometer (PerkinElmer, Waltham, MA, USA) equipped with a Miracle diamond ATR accessory with a 1.8 mm round crystal surface. All spectra were ATR corrected and two single spectra per sample were averaged. The spectra were baseline corrected in the wavenumber range between 4000 and 600 cm^−1^.

### 2.7. Fourier Transform Near-Infrared Spectroscopy

The FT-NIR spectra were obtained from the different surfaces of the wood chips, and from pre-selected sound and dead knots, by an MPA spectrometer (Bruker) equipped with a fibre probe (4 mm measurement diameter) at a resolution of 8 cm^−1^ (32 scans). For every type of wood (e.g., sound and dead knots), a minimum of five samples were selected. Two single spectra of each sample were averaged to minimize the influence of variation on different material surfaces.

### 2.8. Data Analysis

The Unscrambler X 10.3 software (CAMO, Norway) was used for the data analysis. The FT-NIR spectra were managed without data treatment, and also a second derivative method with 17 smoothing points for the presentation of the NIR data was applied. Principal component analysis (PCA) is a linear project method to reduce the multidimensional data to only few orthogonal features (principal components (PCs)) and was used to show the potential of a possible assignment of chemical information of the various wood types.

## 3. Results and Discussion

### 3.1. GC-MS Characterization

Table 1 shows the total amounts of characterized component groups in acetone/water extracts in mg/g of freeze-dried powder of larch wood mixture (heart wood and sapwood), sound knots and dead knots. The amount of total extraction gravimetric yields of the acetone/water solution differed in a range from 3.38 mg/g of dried mixture wood (sapwood and heart wood), 27.13 mg/g of dried sound knotwood and 1.53 mg/g of dried dead knotwood. The hydrophilic extracts of the different wood parts contained various component groups. Based on results of the qualitative analysis all component groups (e.g., carboxylic acids) were found in each material. Nevertheless, there exist high differences in the quantitative results for the group of polyphenols.

**Figure 2:**
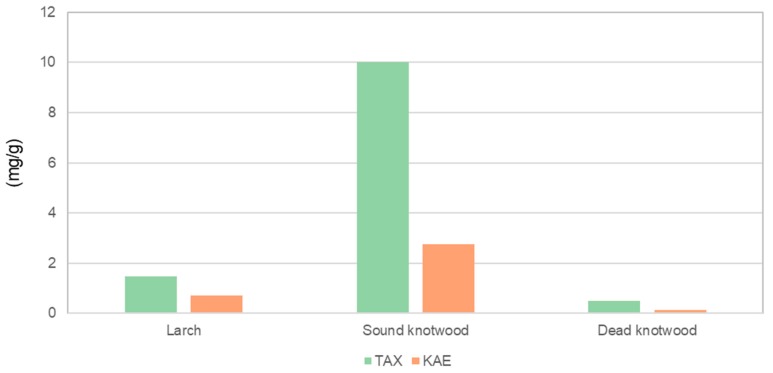
Content and composition of two main phenolic compounds of wood, sound knot and dead knot of larch trees (TAX—taxifolin, KAE—kaempferol).

The taxifolin and kaempferol amounts in acetone/water extracts of the sound knotwood were higher compared to the larch wood and dead knotwood (Figure 2). Both compounds have an antioxidative potency and/or antimicrobial activities [8,12,13]. Compared to the results of other studies, the determined amount of taxifolin and kaemperol were lesser in this study. Zule et al. [6] used only larch heartwood for their study, which has a larger total amount of flavonoids compared to sapwood [15]. The used wood chips for this study were from the outer part of the logs and were mostly sapwood. Nevertheless, the samples of the sound knotwood showed the largest amount of taxifolin and kaempferol compared with the larch wood (Figure 2).

The results of the hexane extracts of the different tree sections showed differences compared to the acetone/water extracts. The amount of total extraction yields from the dead knotwood was the highest for 7.06 mg/g of dried material, followed by the sound knotwood with 5.05 mg/g and larch wood with an amount of 2.46 mg/g of dried materials. Larixol and resin acids (e.g., isopimaric acid) were the main compounds of dead knot and sound knotwood.

### 3.2. Univariate Analyis Method of Different Vibrational Spectrosocpy Methods

**Figure 3:**
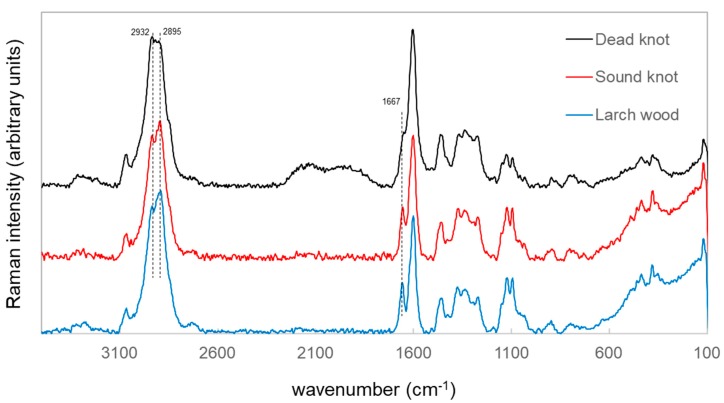
FT-RAMAN spectra of larch wood, sound knotwood and dead knotwood powders, obtained with 1064 nm laser excitation.

The wood powder was also analyzed by an FT-RAMAN spectroscopy method. Based on the results from the GC-MS analysis, small differences for the identification of larch wood, sound and dead knotwood know wood should be characterized. The spectra of the various samples presented almost the same peak profile (Figure 3), while the bands at 2930 and 2895 cm^−1^ changed the ratio to each other, which correspond to the CH groups (e.g., CH_2_ and CH_3_) [20,21] and assigned to different wood compounds (e.g., cellulose and extractives).

Lignin molecules can be observed in the peak at around 1667 and 1660 cm^−1^, corresponding to an unsaturated molecule (C=C) with a carbonyl group (C=O), which overlaps with the vibration of flavonoid aromatic molecules [18,20]. Furthermore, some resin acids of wood have RAMAN vibrational activity in the range of 1666 to 1638 cm^−1^ [20,22]. A slight Raman shift at around 1667 cm^−1^ can be seen by comparison of the spectrum of dead knotwood materials with the other samples. Therefore, a vibration superimposition of lignin, flavonoids and resin acids may have happened in this range. It can be also assumed that the wood extractives are not homogeneously distributed in the different sample types (e.g., wood and knots) and the used samples preparation method did not support a differentiation between different wood and knotwood samples.

**Figure 4:**
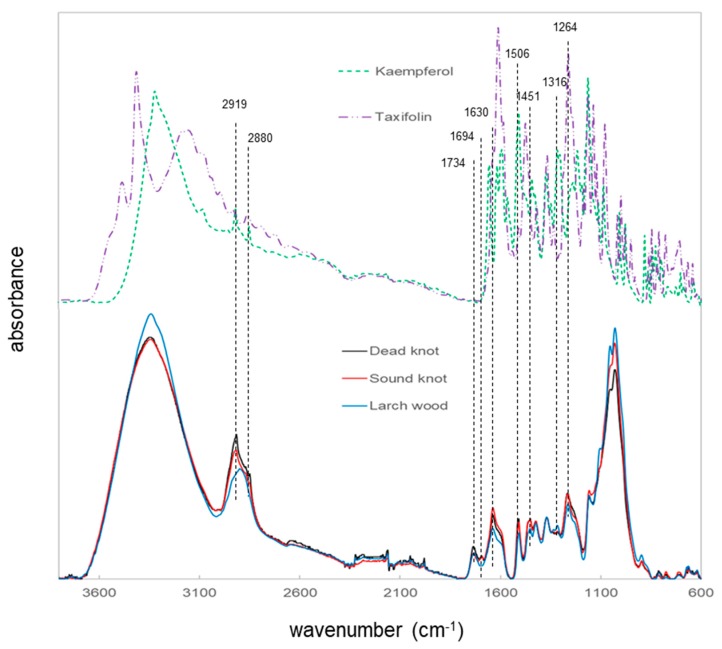
ATR FT-IR spectra of larch wood, sound and dead knotwood as well as kaempferol and taxifolin.

A similar study was run using FT-IR spectroscopy with an ATR unit and the different larch wood types as well as taxifolin and kaempferol as references to analyze the chemical differences (Figure 4), which were found as predominant polyphenols in the GC-MS characterization. The spectrum of larch wood differs significantly in some peak profiles from the knotwood spectra. This tendency is detected for all peaks at 2919, 2880, 1734, 1694, 1630, 1506, 1451, 1316 and 1264 cm^−1^, although the differences cannot be explained in all cases by the consideration of the spectra of taxifolin and kaemperol compounds. The bands around 1736 and 1694 cm^−1^ did not show any IR signal of the references used and can correspond to carbonyl groups in different molecule arrangements [23].

Furthermore, larixol shows vibrational infrared activities in the range of wavenumber between 1750 and 1660 cm^−1^ [24]. Larixol and resin acids were predominantly observed in the different knotwood samples and can correspond to the observed bands in the spectra. The IR absorbance bands at 2919 and 2880 cm^−1^ changed significantly to each other, which correspond to differences in the amount of CH groups (e.g., CH_2_ and CH_3_) [17]. This fact could be assigned to higher amount of bio-polymers in knotwood samples. The infrared spectra depicted that there is a possible differentiation between the larch wood samples and the knotwood samples. The direct measurements on the various wood samples gave more detailed information about the chemical differences compared to the wood powder, which was used for the RAMAN spectroscopy. With respect to these results, a method for classification of different wood samples with vibrational spectroscopy can be further developed. FT-NIR spectroscopy is very often used for this process [17,18,19,20].

**Figure 5:**
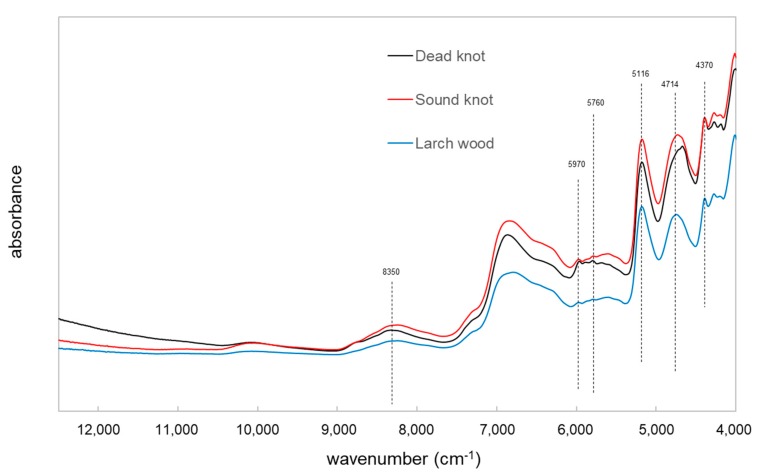
NIR spectra of larch wood, sound and dead knotwood.

**Figure 6:**
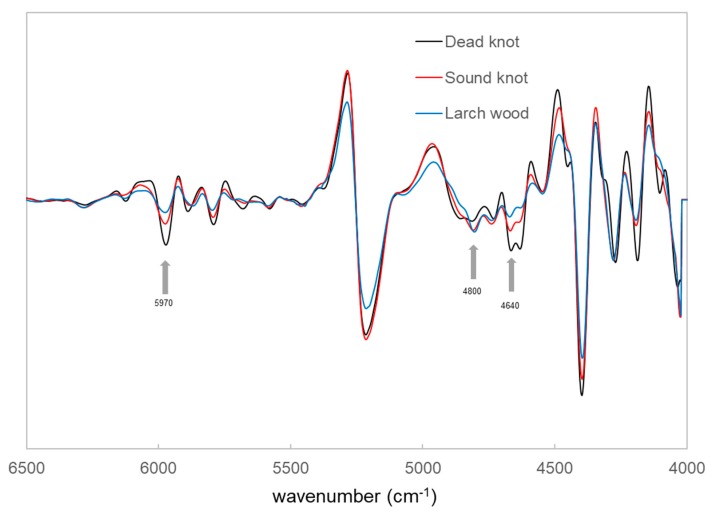
Second derivate FT-NIR spectra of larch wood, sound and dead knotwood.

The chemical information relating to three various larch wood types was obtained by using the FT-NIR spectroscopy (NIRS). Figure 5 shows the original spectra in the region between the wavenumber range 12,500–4000 cm^−1^ of the larch wood, sound knotwood and dead knotwood samples. The original spectra present differences in the wavenumber range between 6000 and 5300 cm^−1^, which are associated with wood extractives mainly from the polyphenols (e.g., taxifolin) [20,25]. The band at around 5970 cm^−1^ is corresponded to the aromatic groups in lignin and wood extractives [26] (Figure 6). Furthermore, a difference in the band at around 4640 cm^−1^ can be observed and could be associated with the combination of C_aryl_-H stretching and C=C stretching [20,27].

For the C-H stretching vibration the NIR spectroscopy presents tree principal bands at around 4370 cm^−1^ the C-H stretch combination, around 5760 cm^−1^ the C-H stretch first overtone, and around 8350 cm^−1^ the C-H stretch second overtone, which have significant correlations for the major C-H stretching bands at 2919 and 2854 cm^−1^ of mid-infrared spectroscopy of lipophilic extractives [28].

The results of the NIR measurement showed the higher absorbance of these bands for the sound and dead knotwood samples. Therefore, it seems that the two types of knots may be distinguished from the larch wood by the univariate analysis of the NIR spectra. Further analysis should be done to show the potential of NIRS for the classification of the various types of wood materials.

### 3.3. Multivariate Data Analysis of FT-NIR Spetra

**Figure 7:**
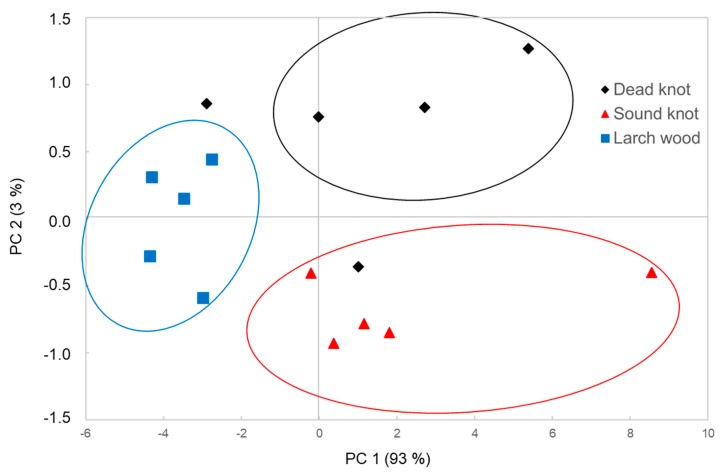
Principal component (PC) analysis score plot of untreated NIR spectra of various larch wood types (e.g., mixture wood, sound and dead knotwood).

The data of the NIRS were used for the classification via principal components analysis (PCA). Figure 7 shows the distribution of two significant factors of the NIRS data. The two principal components explain the highest value of variance within these data and have a great impact on the differentiation of the various wood types used.

Even though the PC 1 explained 93% of the variance, whereas the PC 2 describes only 3% of the variance, the combination of these two components was influenced by the two most important chemical substance groups: polyphenols and lipophilic extractives.

The mixture wood has a lower amount of polyphenols and fatty acids compared to the different knotwood types. The measured samples are clearly separated from the other materials (Figure 6). The sound knotwood samples presented a higher amount of taxifolin and kaempferol, whereas a lower amount of larixol than in the dead knotwood samples can be observed. Therefore, the score plot of the PCA shows also differences between sound and dead knotwood samples. Nevertheless, there are some areas without direct assignment of the samples (e.g., dead knotwood samples). The NIR measurements were performed on different surfaces from various selected samples of the larch wood, sound and dead knotwood. It can be assumed that the wood extractives distribution is not homogeneous. Therefore, some areas have a high amount of wood extractives, whereas some areas have a low amount.

**Figure 8:**
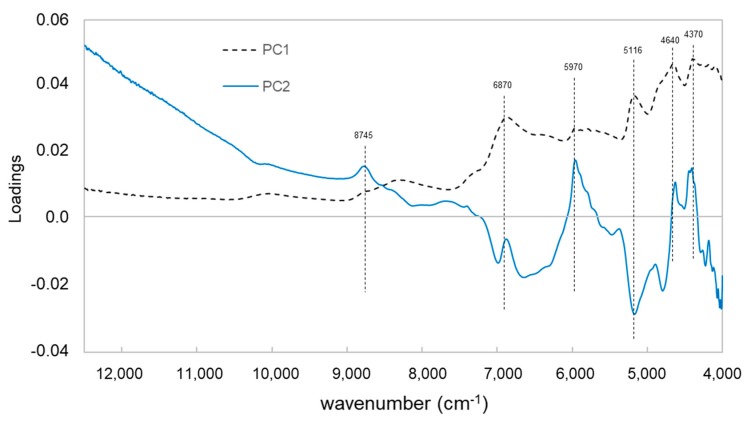
Loadings of the first two principal components (PCs) of near infrared spectra of various wood types.

The loadings of the PC 1 state high positive values for the wavenumbers 6870, 5970, 4640 and 4370 cm^−1^, which are mainly corresponded to the phenolic compounds in wood extractives (Figure 8). The results of principal component analysis showed that the loadings of the PC 2 are contrary to the loadings of the PC 1 at the same NIR band at around 5116 cm^−1^ (water band of NIR signal), while the loadings of the PC 2 contribute to other wood extractives (e.g., lipophilic compounds) with lower moisture content within the samples.

## 4. Conclusions

Differences in wood extractives from various resources of larch wood, sound and dead knotwood were observed by a GC-MS method. Sound knotwood can provide a greater extraction yield than dead knotwood and larch wood, mainly sapwood with a small amount of heartwood, from the outer part of the logs, while more resin acids and larixol can be extracted from dead knotwood samples compared to the sound knotwood and larch wood samples.

The results from the GC-MS were used to analyze three various spectroscopy methods for the potential of the characterization of wood extractives and identification of the three wood types. With all vibrational spectroscopy used, chemical differences between the wood and knotwood were observed and can be used to fulfil various research tasks. Moreover, the methods of multivariate statistics were applied to analyze materials by using the score plot and loadings of the PC analysis. These results demonstrate that a classification of various wood tissues based on the different chemical components (e.g., polyphenols and lipophilic substances) is possible with fast, non-destructive measurement methods. The current unused wood chips for material use can be separated into different fractions, where the extraction yields of the target substance (e.g., taxifolin) are higher compared to the other fractions. This method may serve as basis to establish guidelines for quality assurance control systems of this new approach for material use to obtain the huge potential of bio-based products for innovative applications and for further efforts in the upscale from laboratory to industrial conditions.

## Figures and Tables

**Table 1:** Main component groups in acetone/water extracts of different larch wood materials by GC-MS.

Component Groups	Larch Wood Mixture (mg/g)	Sound Knotwood (mg/g)	Dead Knotwood (mg/g)
Carboxylic acids	0.026	0.139	0.011
Phenolic acids	0.116	1.666	0.081
Sugar	0.206	0.472	0.045
Fatty acids	0.140	0.483	0.047
Resin acids	0.079	0.186	0.014
Polyphenols	2.726	23.766	1.305
Sterols	0.016	0.235	0.011
